# Supplementary material for: Temporal pattern separation in hippocampal neurons through multiplexed neural codes
Source: PLoS Comput Biol. 2019 Apr 22;15(4):e1006932. doi: 10.1371/journal.pcbi.1006932 (PMC6476466; doi:10.1371/journal.pcbi.1006932)
Supplement: S1 Appendix — (PDF) [file pcbi.1006932.s001.pdf]

S1 Appendix

Authors: Antoine D. Madar, Laura A. Ewell, Mathew V. Jones

This document provides supplementary figures, tables and a discussion on how to interpret the similarity and burstiness metrics used in the article "Temporal pattern separation in hippocampal neurons through multiplexed neural codes".

1 – How to interpret similarity metrics

Measuring the similarity between two spike trains is a difficult problem [1, 2], and many methods have been designed to this effect, each with a set of pros, cons and assumptions [2-6]. We could not be exhaustive but, in order to span a large range of potential neural codes, we used four different similarity metrics: R (Pearson's correlation coefficient), NDP (Normalized Dot Product), SF (Scaling Factor) and SPIKE (1 - SPIKE distance). (Note: 'metrics' is used with a loose sense throughout the paper). Their pros, cons and assumptions are detailed in this section and summarized in **Table 1** of the article.

Binned vs binless metrics

We used two different kinds of similarity metrics: 1) binned metrics (R, NDP, SF) consider a spike train as a list X of spike counts X_i (with X_i the number of spikes in time bin i of a given duration τ_w) and thus assume a neural code where spike counts are the basic informative feature; 2) a binless metric SPIKE that considers a spike train as a list of spike times and thus assumes a neural code where the time relative to the start of the given spike train is the basic informative feature.

Although R has already been used to measure the similarity between spike trains [2, 7], none of the binned metrics we used were designed with spike trains in mind and thus have properties that might not be well suited for the study of spike trains. For instance, R, NDP and SF have the drawback of considering all X_i (i.e. binned spike counts) as independent observations, which may not be a realistic assumption. Moreover, they assume spike trains are related linearly, or that they belong to a Euclidean space. Binless metrics like SPIKE avoid these limitations [8].

Another limitation of binned metrics as implemented here (and this is also true for Compactness and Occupancy) is that they are subject to edge effects: small shifts of the bins can include or exclude spikes, which would lead to differences in the similarity measure. However, the fact that marginally different binsizes (e.g. 5 ms vs 10 ms) lead to similar results (in other words, similarity measures as a function of τ_w does not show spurious values: see Figure 3A-C, 6A, 7D, 8) suggests that edge effects do not impair our conclusions.

Differences between binned metrics

<u>R vs NDP and SF: epistemological differences</u>

The Pearson's correlation coefficient R was designed by early statisticians as a tool to quantify the strength and direction of a linear regression [9]. R thus considers two spike trains X and Y as variables, with X_i and Y_i the observations, and assesses, across all i, whether X_i and Y_i tend to vary in a similar fashion (**Figure 2B**).

On the other hand, NDP and SF are metrics coming from the fields of Euclidean geometry and linear algebra. In contrast to R, they explicitly consider spike trains as vectors, with each time bin i corresponding to a coordinate, or dimension, in an N-dimensional space (N being the total number of bins). NDP measures the angle between two vectors, and SF is based on vector norms (**Figure 2C**).

R, NDP and SF are not equivalent

Although they have been designed for different purposes, both R [7] and NDP [10, 11] have been used to measure the similarity between neuronal activity patterns, including spike trains. In this context, NDP has sometimes loosely been called a measure of correlation [10, 12]. What difference is there between R and NDP then, especially in terms of the assumed neural code? A comparison of equation 1 and 2 (**Methods**) reveals that R is actually an NDP, but of baseline-subtracted vectors. In other words, R does not consider spike trains as raw spike count vectors but as vectors of deviation from their respective mean spike count. This ensures that R is an optimal estimator of correlation [9] but it has the inconvenience of not conserving the angular relationship between X and Y, making NDP necessary if one wants to evaluate pattern separation as an orthogonalization process. Moreover, using baseline-subtracted vectors also means that R considers common silent periods (i.e. $X_i = Y_i = 0$) as correlated, which is an assumption on the neural code that has been considered unrealistic by some [2]. In contrast, NDP excludes those empty bins and thus assumes a neural code where common periods of inactivity are irrelevant.

Comparing equations 1, 2 and 3 (**Methods**), the relationships between R, NDP and SF appear not trivial and not linear. To further evaluate the relationship between R, NDP and SF, we compared the values of each similarity metrics for all pairs of a set of simulated spike trains. This set contained the $3^6 = 729$ possible spike count vectors made of six bins, with each bin containing 0, 1 or 2 spikes (some examples are displayed in **Figure 2A**). This analysis confirmed that the three metrics are not equivalent and provides an intuition on what each metric represents (**Figure S1A**).

Interestingly, even though R, NDP and SF are not linearly related in principle, our measures of R and NDP between experimentally recorded spike trains have a near linear relationship, as opposed to R and SF (**Figure S1B**). This correlation is likely due to our choice of input spike trains as well as to biological constraints (e.g. refractory period) forcing the temporal structure of output spike trains to vary only along certain dimensions. It is thus reflective of the actual computations performed in the DG and shows that although R and NDP carry different sets of assumptions about the neural code, spike trains recorded in GCs vary mostly along properties that both R and NDP are sensitive to.

R and NDP vs SF: sensitivity to individual firing rate differences and dependency on pairwise firing rate levels

To interpret R, NDP and SF and their different assumed neural codes, it is important to understand their relationship with the firing rate. Studying the impact of firing rate on similarity metrics can however mean different things.

First, a similarity metric can be sensitive to differences of firing rate between two spike trains X and Y. For instance, if $X = kY$ (with k an integer > 1), that means that X and Y have spikes in exactly the same bins (i.e. X and Y have the same temporal structure) but that Y has a higher firing rate. In such a case, both R and NDP would consider X and Y as perfectly similar (see example #2 in **Figure 2A-C**). In contrast, SF is designed to extract 1/k and thus strongly

picks up on differences of firing rate. On the other hand, if X and Y have the same firing rate but that their spikes are in different bins (different temporal structure), R and NDP will consider those spike trains completely dissimilar, and SF will see them as perfectly similar (see example #1 in **Figure 2A-C**). Other cases, where X and Y have spikes in the same time bins but only some bins have a different number of spikes (e.g. example #3 in **Figure 2A-C**) reveal that R and NDP are not totally insensitive to differences of firing rate when those are not proportional, but overall these two metrics are mostly sensitive to binwise synchrony, unlike SF (**Figure S2**).

Second, similarity metrics can be influenced by the overall firing rate level of a pair of spike trains (for instance measured as the geometric mean of the firing rate of X and Y). Whether an ideal similarity metric should be independent of the firing rate in such a way is debatable, and finding one is not trivial [2, 13], yet it is generally seen as an undesirable property [2] because it makes the comparison of similarity values between two pairs of spike trains more challenging to interpret [1]. Indeed, for spike trains X and Y with random spike times, the probability of spikes occurring close in time will intuitively be higher if X and Y have higher rates [14], even though their temporal structure would be conserved.

Because R is often used to measure spike train correlations [1], its relationship to pairwise firing rate levels has already been studied. It was for instance demonstrated that, when X and Y are two Poisson spike trains with the same rate λ, the normalization factor and the use of variables centered on their mean (which is, in the case of spike trains, a linear function of the firing rate) makes R mathematically independent of λ [14]. However, in the general case, R has still a complex relationship with the firing rates of both spike trains [13].

To get a better sense of this relationship for R, and study the effect of pairwise firing rate levels on NDP and SF as well, we considered two cases: 1) proportional firing rate increases, and 2) additive firing rate increases.

In the first case, we considered two spike trains A and B such that A = kX and B = kY (with k an integer > 1). Some metrics aiming to measure the similarity between spike train in terms of temporal structure, like the correlation index, have been shown to result in higher values for the (A, B) pair compared to the (X, Y) pair [2]. However, in this case, R, NDP and SF values would be the same for both pairs (A, B) and (X, Y): thanks to the denominator in their respective formula, all three are insensitive to proportional increases in firing rates.

In the second case, we used the set of 729 6-bin spike count vectors described above and generated new sets of related vectors with higher firing rates, such that for each spike train X of the original set there was a vector A in the new set with $A_i = X_i + k$ when $X_i > 0$, and $A_i = X_i$ otherwise. In other words, the rate was additively (not proportionally) increased while preserving the temporal structure of the initial spike trains and maintaining constant the difference in the binwise number of spikes between spike trains (e.g. for k = 5, if X = [010101] and Y = [020202], then A = [060606] and B = [070707]. For both pairs, the difference is [010101].). Our analysis shows that such an increase in the firing rate can affect all three metrics, but in different ways (**Figure S1C**): NDP and R can increase or decrease, and on average do not change much, whereas SF systematically increases. A larger increase of the firing rate leads to more variability of the effect size for NDP and R whereas the effect monotonically increases for SF.

SF is thus clearly dependent on pairwise firing rate levels, and R and NDP can be affected somewhat as well. Thus, to directly assess the variability of specific spike train features across a set of spike trains (i.e. individual firing rate, Compactness and Occupancy) without contaminating interpretations with consideration of pairwise firing rate levels (especially in the case of SF), we used additional metrics detailed in **Methods – dispersion metrics**.

Temporal resolution of binned metrics

As binned metrics, R, NDP and SF are dependent on the binsize, i.e. a specified temporal resolution τ_w. In other words, in equations 1 to 3, X_i and Y_i are functions of τ_w. Temporal resolution logically influences the value of metrics measuring similarity in terms of the temporal structure of spike trains, such as R [1] or NDP. However it also influences SF: even though SF is sensitive to differences in firing rate, it is designed to be sensitive to differences in binwise firing rate, which makes it also sensitive to differences in burstiness even when the firing rate is constant (e.g. **Figure 4B** and **Figure S2**).

In **Figure 3**, we evaluated the influence of τ_w on our results by varying it between 5 ms and 1000 ms. Note that because our spike trains are two-seconds long, using $\tau_w = 2000$ ms would mean the spike trains can be seen as variables with only one observation, or as unidimensional vectors (i.e., scalars) that can only vary by their norm (i.e., firing rate). In this case, R is meaningless, NDP is necessarily 1, which indicates collinearity and SF would directly assess the difference in total spike number (i.e. overall firing rate) between the two spike trains.

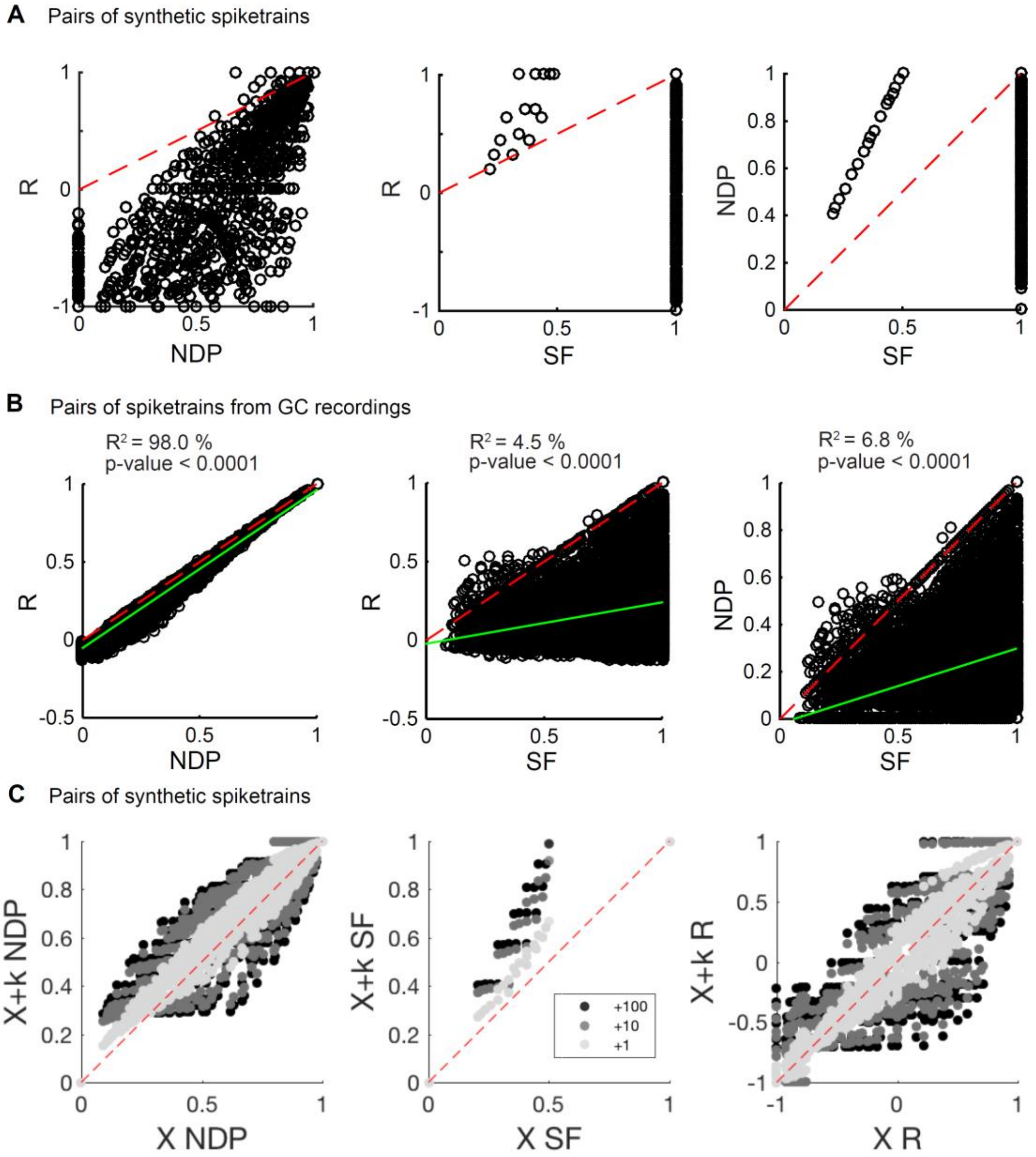

Figure S1 (related to Figure 2). **R, NDP and SF are not equivalent.**

(A) Relationships between these three similarity metrics (R, NDP and SF) computed between the 3^6 possible spike count vectors with six bins and that can have 0, 1 or 2 spikes per bin (All pairs combinations = 499,500 data points).

(B) Relationships between these three similarity metrics for the 124,950 pairs of spike trains from the 102 experimental GC recording sets. Green lines correspond to a linear regression (R^2 and p-value in each panel). Note that although R and NDP are well correlated in our experimental data (R^2>0.95), there is not a linear relationship between R and NDP in theory.

(**C**) Impact of non-proportional firing rate increase on similarity values for all three metrics. For each 6-bin spike count vector X (same as in A), a vector A was generated such that for each bin i with at least one spike, $A_i = X_i + k$, with k = 1, 10 or 100, and $A_i = X_i$ when $X_i = 0$ (e.g. for k = 1, if X = [010101] and Y = [020202], then A = [020202] and B = [030303]. For both pairs, the difference is [010101]). NDP, SF and R values were computed for all pairs in each set of vectors, and the values of the initial set (x-axis) was compared to the values of the X+k set (y-axis). Data points away from the identity line (red) demonstrate an effect of the firing rate increase.

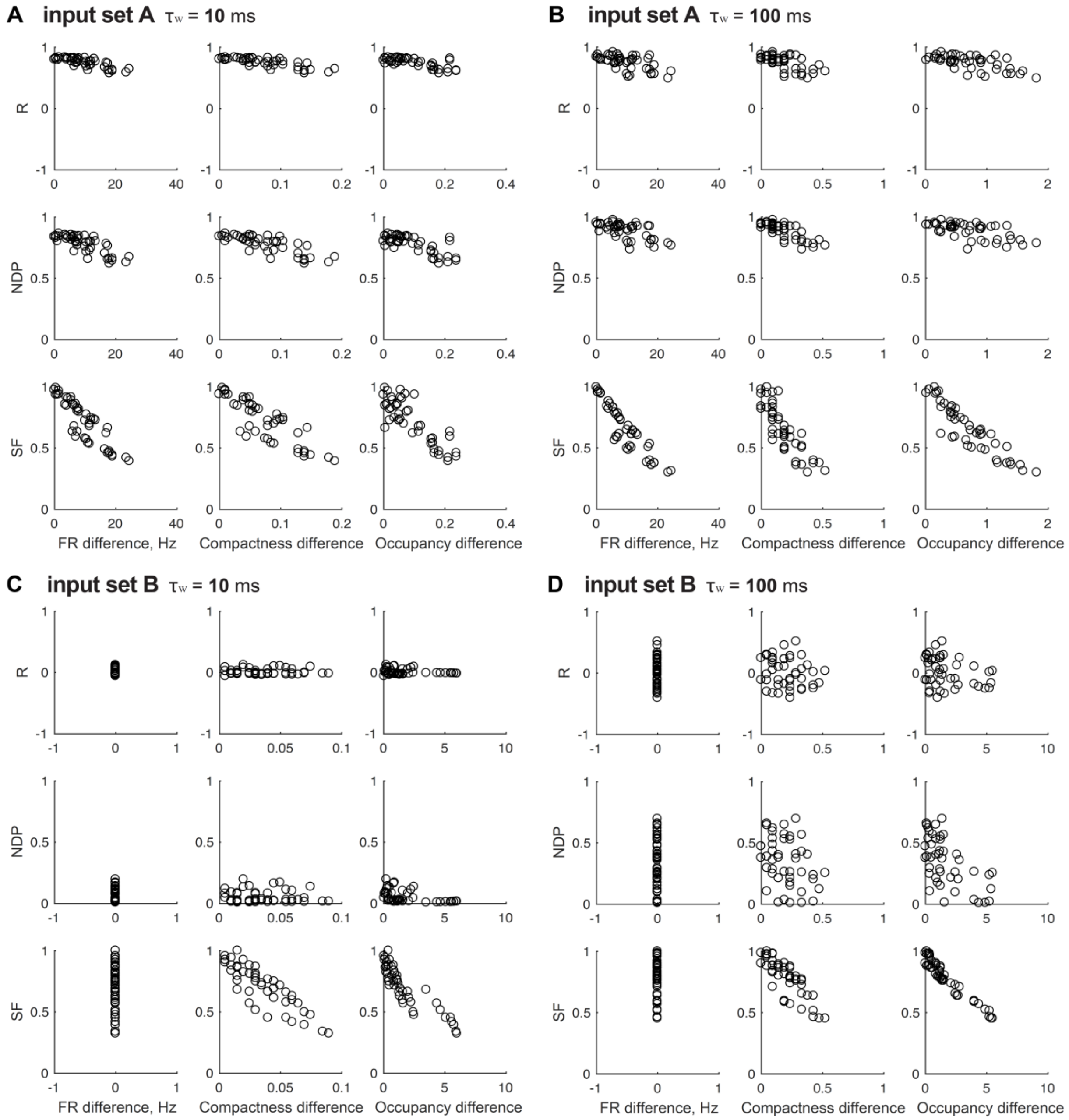

Figure S2 (related to Figure 4)**. Influence of firing rate and burstiness differences on binned similarity metrics.**

Data points correspond to single pairs of input trains from input set A (**A-B**) or input set B (**C-D**). The similarity between two spike trains (R, NDP or SF), their difference of Compactness (proportion of occupied bins), and their difference in Occupancy (average number of spikes per occupied bins) were measured using binning windows of 10 ms (**A, C**) or 100 ms (**B, D**). When FR is constant across spike trains, Compactness and Occupancy differences are direct measures of burstiness differences, given a certain time scale. As intuited from Figure 2A-C, SF is very sensitive to variations in FR, Compactness and Occupancy between spike trains, whereas R and NDP are only mildly influenced at best (and mostly at larger time scales), by such variations.

2 – Burstiness metrics

Cell burstiness vs spike train burstiness

To assess the tendency of a neuron to fire bursts, we computed the Kullback-Leibler divergence $D_{KL}(M||P)$ of the ISI distribution in a set of output spike trains from the ISI distribution of a Poisson process. $D_{KL}(M||P)$ is an asymmetric, information theory-based measure of the inefficiency of the assumption that the distribution of ISI M is a distribution P (Poisson, in our case), and thus can be interpreted as the divergence of one probability distribution from another. Note that a train with regularly spaced spikes would diverge from Poissonness without being bursty, but that natural spike trains are rarely regular, and accordingly none of our recordings showed a regular ISI distribution, suggesting that $D_{KL}(M||P)$ is a good indirect indicator of burstiness.

The D_{KL} approach above has the advantage of being independent of the firing rate, but has two limitations. First it is parametric, based on the nonconsensual opinion that a Poisson process is the least bursty of processes [15]. Second, because it is based on the comparison of ISI distributions, it requires a large sample of ISIs to be accurate and thus cannot be used to measure the burstiness of a single two second spike train.

In order to assess the burstiness of single spike trains, we designed two binned metrics: Compactness and Occupancy. We used binned metrics to avoid the difficult problem of burst detection that generally requires some arbitrary threshold to define what is considered a burst [16]. Unlike D_{KL} these metrics are not independent of the firing rate, but together they allow distinguishing between burstiness and firing rate (see below).

Compactness and Occupancy: complementary relationships to the firing rate allow disambiguating between burstiness and lack of sparseness

It is unclear whether the concept of burstiness should be independent or not of the firing rate (for instance, should we consider a spike train with 100 spikes clustered in the same 10 ms bin as bursty as a spike train with just 10 spikes in a single bin?), but, as explained above for similarity metrics, a dependency of burstiness metrics on the firing rate can make interpretations more challenging. Both Compactness and Occupancy are dependent on the firing rate, but in different ways, which resolves the ambiguity: 1) Compactness does not provide information on the firing rate per se, and is only related to the firing rate in the sense that, in the case of Poisson-like spiking, higher rates lead to a higher chance of occupying a large number of bins. Thus, a spike train with just one spike will be considered as compact as a spike train with a large number of spikes all clustered into one bin. In contrast, the Occupancy is directly related to the number of spikes and thus to the firing rate of a given spike train (Indeed, when considering a time bin as long as the full duration of a spike train, i.e. two seconds in our case, the Occupancy is exactly proportional to the firing rate.). Occupancy would thus distinguish between a sparse spike train with just one spike (Occupancy = 1) and a spike train with 10 spikes clustered in one bin (Occupancy = 10). 2) On the other hand, spike trains with different firing rates can have the same Occupancy (e.g. if all spikes are distributed in different bins, Occupancy = 1 regardless of the number of spikes) but would be differentiated by their level of Compactness (100 distributed spikes would be considered much less compact than a spike train with just one spike). Therefore, when assessing pairs of spike trains with different firing rates, Compactness and Occupancy provide complementary information that allows determining whether one spike train is unambiguously burstier or simply has a higher firing rate (see **Table S1**).

Table S1. (related to Figure 7) **How to interpret Compactness and Occupancy in terms of burstiness and sparseness when comparing two spike trains?**
The table shows what it means when *Compactness* (C) is increased, stays constant or decreases while *Occupancy* (O) increases, stays constant or decreases. We distinguish the cases when the mean firing rate (FR) is constant (FR absolute difference = 0) or different (FR abs diff > 0). The second spike train is either unambiguously burstier (B+) or less bursty (B-), has a greater FR (F+) or is sparser (F-), or a combination of those (i.e. B+F+ or B-F-). In each case, we provide an example (4-dimensional vectors of spike counts) when the conditions can coexist (NA means not applicable). Notice that the resulting table of examples is centrally antisymmetric: the central cell is invariant and every other cell around the center is the reverse image of the 180° opposite cell (e.g. B+, 0101 → 0200 as opposed to B-, 0200 → 0101).

**C** \ **O**		↗	–	↘
↗	FR abs diff = 0	**B+**<br>0101 → 0200	NA	NA
	FR abs diff > 0	**B+F+**<br>0111 → 0501	**F-**<br>0202 → 0200	**F-**<br>0222 → 0101
–	FR abs diff = 0	NA	2020 → 0202	NA
	FR abs diff > 0	**F+**<br>0101 → 0202	NA	**F-**<br>0202 → 0101
↘	FR abs diff = 0	NA	NA	**B-**<br>0200 → 0101
	FR abs diff > 0	**F+**<br>0101 → 0222	**F+**<br>0200 → 0202	**B-F-**<br>0501 → 0111

References

1. Cohen MR, Kohn A. Measuring and interpreting neuronal correlations. Nature neuroscience. 2011; 14(7): 811. doi.

2. Cutts CS, Eglen SJ. Detecting pairwise correlations in spike trains: an objective comparison of methods and application to the study of retinal waves. J Neurosci. 2014; 34(43): 14288-303. doi: 10.1523/jneurosci.2767-14.2014.

3. Lyttle D, Fellous JM. A new similarity measure for spike trains: sensitivity to bursts and periods of inhibition. J Neurosci Methods. 2011; 199(2): 296-309. doi: 10.1016/j.jneumeth.2011.05.005.

4. Kreuz T. Measures of spike train synchrony. Scholarpedia [Internet]. 2011; 6(10). Available from: http://www.scholarpedia.org/article/Measures_of_spike_train_synchrony.

5. Rusu CV, Florian RV. A new class of metrics for spike trains. Neural Computation. 2014; 26(2): 306-48. doi.

6. Satuvuori E, Kreuz T. Which spike train distance is most suitable for distinguishing rate and temporal coding? Journal of neuroscience methods. 2018; 299: 22-33. doi.

7. Yim MY, Hanuschkin A, Wolfart J. Intrinsic rescaling of granule cells restores pattern separation ability of a dentate gyrus network model during epileptic hyperexcitability. Hippocampus. 2014. doi: 10.1002/hipo.22373.

8. Aronov D, Victor JD. Non-Euclidean properties of spike train metric spaces. Physical Review E. 2004; 69(6): 061905. doi.

9. Stanton JM. Galton, Pearson, and the peas: A brief history of linear regression for statistics instructors. Journal of Statistics Education. 2001; 9(3). doi.

10. Schreiber S, Fellous JM, Whitmer D, Tiesinga P, Sejnowski TJ. A new correlation-based measure of spike timing reliability. Neurocomputing. 2003; 52-54: 925-31. doi: 10.1016/s0925-2312(02)00838-x.

11. Aimone JB, Wiles J, Gage FH. Computational influence of adult neurogenesis on memory encoding. Neuron. 2009; 61(2): 187-202. doi: 10.1016/j.neuron.2008.11.026.

12. Dieni CV, Panichi R, Aimone JB, Kuo CT, Wadiche JI, Overstreet-Wadiche L. Low excitatory innervation balances high intrinsic excitability of immature dentate neurons. Nat Commun. 2016; 7: 11313. doi: 10.1038/ncomms11313.

13. Amari S-i. Measure of correlation orthogonal to change in firing rate. Neural computation. 2009; 21(4): 960-72. doi.

14. de la Rocha J, Doiron B, Shea-Brown E, Josic K, Reyes A. Correlation between neural spike trains increases with firing rate. Nature. 2007; 448(7155): 802-6. doi: 10.1038/nature06028.

15. Gourevitch B, Eggermont JJ. A nonparametric approach for detection of bursts in spike trains. J Neurosci Methods. 2007; 160(2): 349-58. doi: 10.1016/j.jneumeth.2006.09.024.

16. Cotterill E, Charlesworth P, Thomas CW, Paulsen O, Eglen SJ. A comparison of computational methods for detecting bursts in neuronal spike trains and their application to human stem cell-derived neuronal networks. J Neurophysiol. 2016; 116(2): 306-21. doi: 10.1152/jn.00093.2016.
