# Supplementary figures and images for: Temporal pattern separation in hippocampal neurons through multiplexed neural codes
Source: PLoS Comput Biol. 2019 Apr 22;15(4):e1006932. doi: 10.1371/journal.pcbi.1006932 (PMC6476466; doi:10.1371/journal.pcbi.1006932)

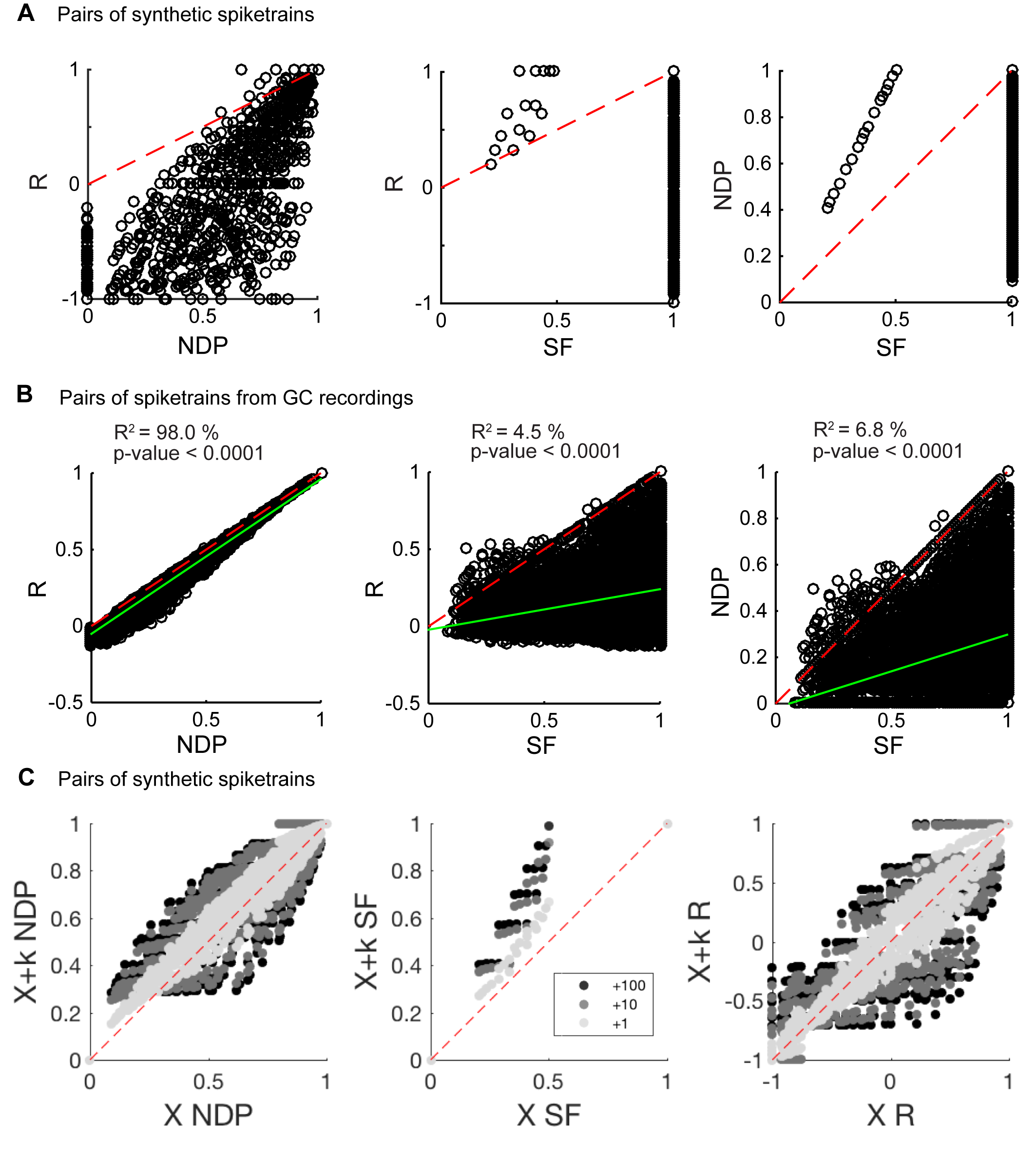

Supplement: S1 Fig — R, NDP and SF are not equivalent. (A) Relationships between these three similarity metrics (R, NDP and SF) computed between the 36 possible spike count vectors with six bins and that can have 0, 1 or 2 spikes per bin (All pairs combinations = 499,500 data points). (B) Relationships between these three similarity metrics for the 124,950 pairs of spike trains from the 102 experimental GC recording sets. Green lines correspond to a linear regression (R2 and p-value in each panel). Note that although R and NDP are well correlated in our experimental data (R2>0.95), there is not a linear relationship between R and NDP in theory. (C) Impact of non-proportional firing rate increase on similarity values for all three metrics. For each 6-bin spike count vector X (same as in A), a vector A was generated such that for each bin i with at least one spike, Ai = Xi + k, with k = 1, 10 or 100, and Ai = Xi when Xi = 0 (e.g. for k = 1, if X = [010101] and Y = [020202], then A = [020202] and B = [030303]. For both pairs, the difference is [010101]). NDP, SF and R values were computed for all pairs in each set of vectors, and the values of the initial set (x-axis) was compared to the values of the X+k set (y-axis). Data points away from the identity line (red) demonstrate an effect of the firing rate increase. (TIF) [file pcbi.1006932.s002.tif]

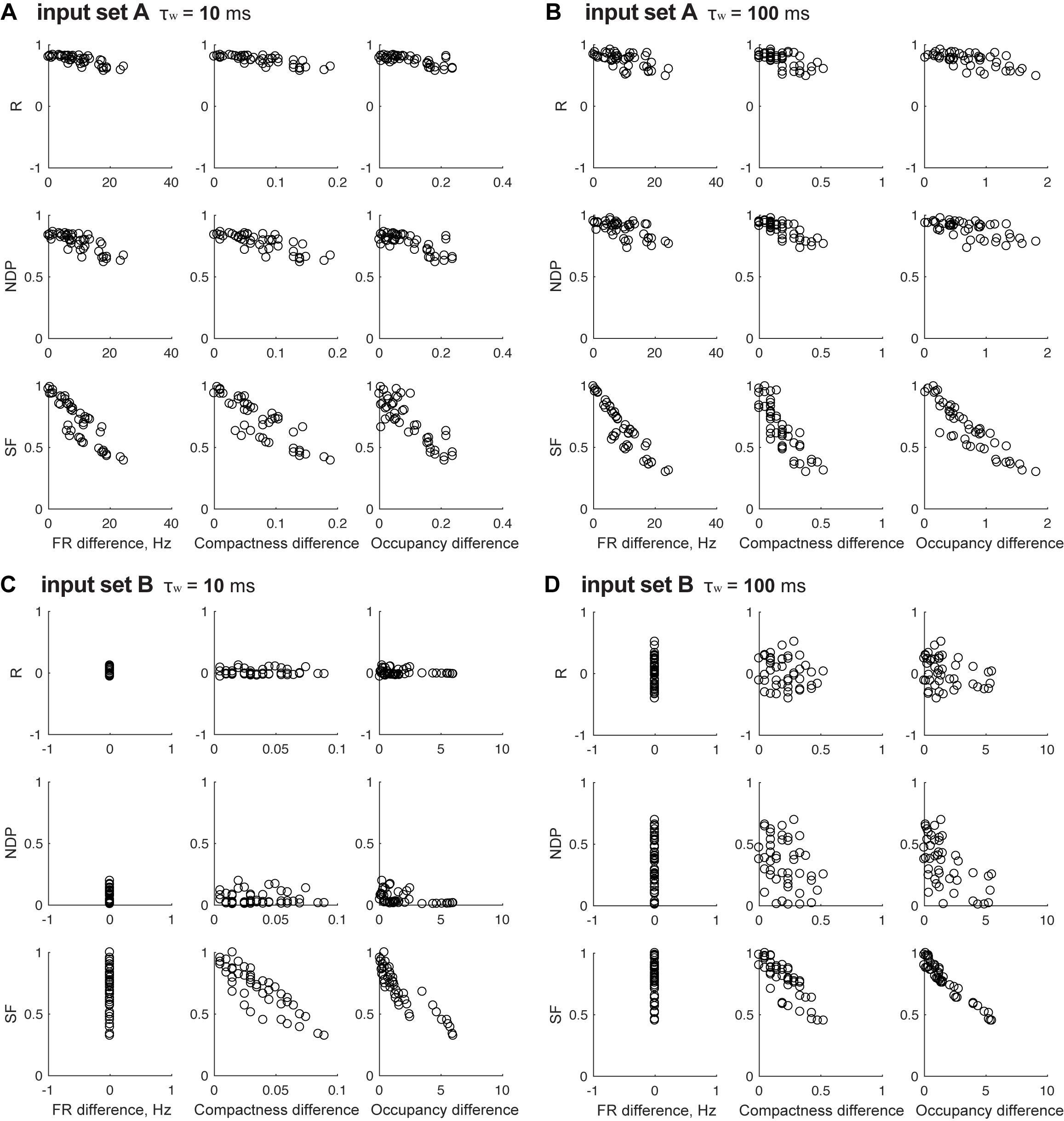

Supplement: S2 Fig — Influence of firing rate and burstiness differences on binned similarity metrics. Data points correspond to single pairs of input trains from input set A (A-B) or input set B (C-D). The similarity between two spike trains (R, NDP or SF), their difference of Compactness (proportion of occupied bins), and their difference in Occupancy (average number of spikes per occupied bins) were measured using binning windows of 10 ms (A, C) or 100 ms (B, D). When FR is constant across spike trains, Compactness and Occupancy differences are direct measures of burstiness differences, given a certain time scale. As intuited from Fig 2A–2C, SF is very sensitive to variations in FR, Compactness and Occupancy between spike trains, whereas R and NDP are only mildly influenced at best (and mostly at larger time scales), by such variations. (TIF) [file pcbi.1006932.s003.tif]

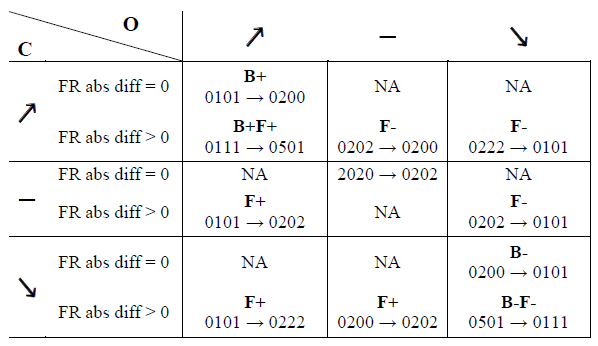

Supplement: S1 Table — How to interpret Compactness and Occupancy in terms of burstiness and sparseness when comparing two spike trains? The table shows what it means when Compactness (C) is increased, stays constant or decreases while Occupancy (O) increases, stays constant or decreases. We distinguish the cases when the mean firing rate (FR) is constant (FR absolute difference = 0) or different (FR abs diff > 0). The second spike train is either unambiguously burstier (B+) or less bursty (B-), has a greater FR (F+) or is sparser (F-), or a combination of those (i.e. B+F+ or B-F-). In each case, we provide an example (4-dimensional vectors of spike counts) when the conditions can coexist (NA means not applicable). Notice that the resulting table of examples is centrally antisymmetric: the central cell is invariant and every other cell around the center is the reverse image of the 180° opposite cell (e.g. B+, 0101 → 0200 as opposed to B-, 0200 → 0101). (TIF) [file pcbi.1006932.s004.tif]
